# The role of indole derivative in the growth of plants: A review

**DOI:** 10.3389/fpls.2022.1120613

**Published:** 2023-01-16

**Authors:** Ping Sun, Yuanqin Huang, Xiaoyan Yang, Anjing Liao, Jian Wu

**Affiliations:** National Key Laboratory of Green Pesticide, Key Laboratory of Green Pesticide and Agricultural Bioengineering, Ministry of Education, Guizhou University, Guiyang, China

**Keywords:** indole derivatives, plant growth regulators, plant immune inducers, biological stress, abiotic stresses, mechanisms of action

## Abstract

Indole compounds with their unique properties of mimicking peptide structures and reversible binding to enzymes are of great exploitative value in the regulation of plant growth. They stimulate root and fruit formation and activate the plant’s immune system against biotic and abiotic factors harmful to the plant. Analysis of target recognition, receptor recognition, key activation sites and activation mechanisms of indoles in plant to enhance crop growth or disease resistance is a crucial step for further developing compounds as plant growth regulators and immune inducers. Therefore, this review focused on the mechanism of action of indoles in regulating plant growth and enhancing plant resistance to biotic and abiotic stresses.

## Introduction

Synthesized or extracted artificially, plant growth regulators, also known as phytohormones, possess a physiological effect that is comparable to that of natural plant hormones. Within plants, they bind to hormone receptors in plant cells to form complexes that recognize hormone signals, which in turn trigger a series of physiological and biochemical reactions in the plant, ultimately leading to morphological changes in the plant (Nikonorova et al., 2021). Plant immune inducers act as a catalyst to activate the immune system, making it better defend against agricultural pests and diseases. Within plants, the induction of salicylic acid (SA) and jasmonic acid (JA) biosynthesis can be induced (Gozzo and Faoro, 2013), resulting in the hypersensitive reaction (HR) of the plant cell, which leads to its death to protect the plant from further colonization of pests and diseases (Chen et al., 2014).

**Figure 1:**
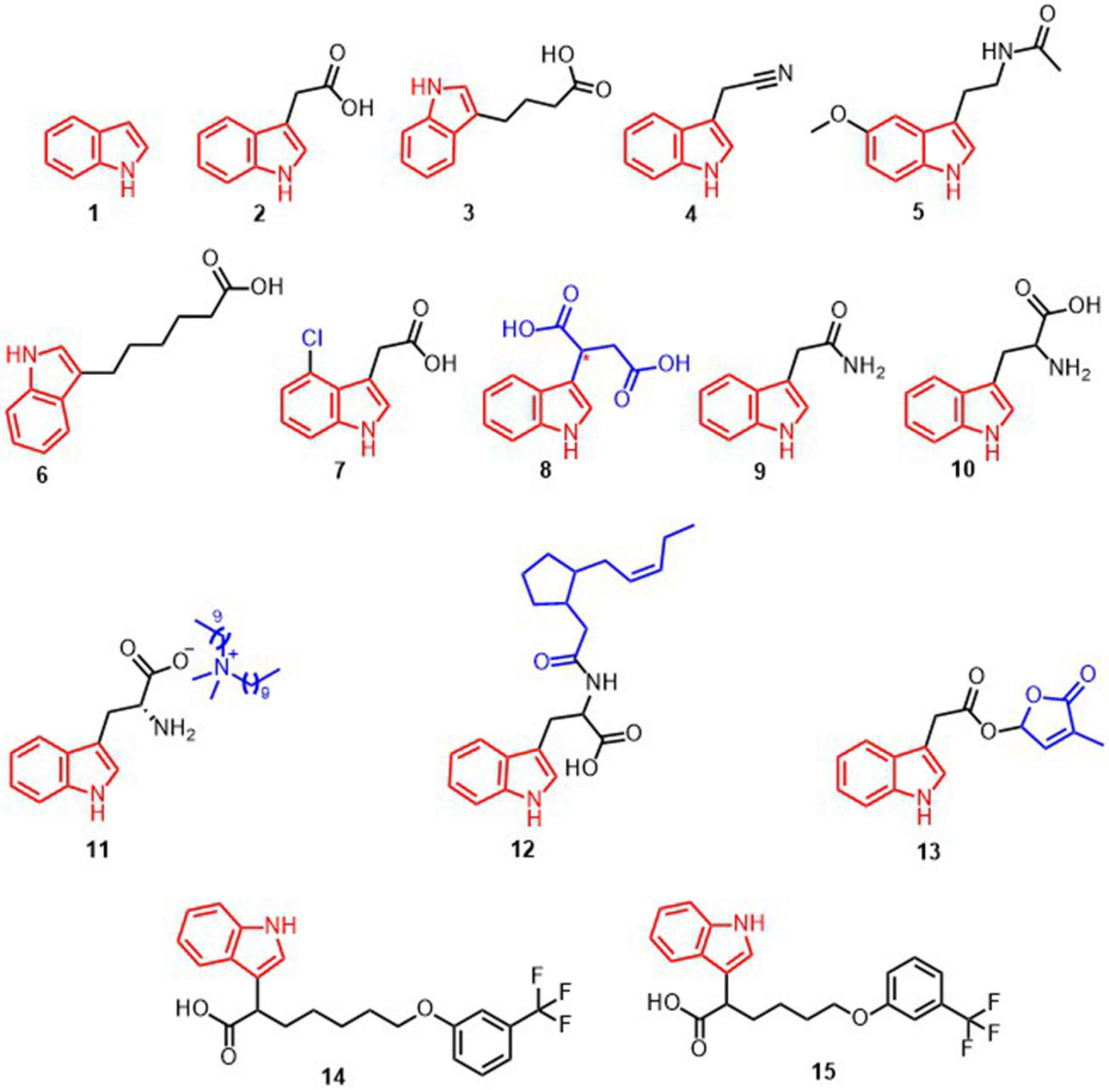
Structural formula of compounds 1-15.

Indole **1** (**Figure 1**) is a significant structure in drug discovery, as it functions as a scaffold for various receptors (de Sá Alves et al., 2009; Zhang and Chen, 2014b). Indole-based compounds, such as indoleacetic acid (IAA) **2** (**Figure 1**) (Chen et al., 2020) and indole-3-butyric acid (IBA) **3** (**Figure 1**) (Li et al., 2018), are commonly used as plant growth regulators in agricultural settings. Indole-3-acetonitrile (IAN) **4** (**Figure 1**) has been documented to be an effective plant growth regulator, with its efficacy being ten-fold that of IAA. Additionally, it is converted to IAA with growth-regulating effects in plants (Osborne, 1952; Sun et al., 2018). The emergence of indole compounds has revealed a multitude of indole derivatives that can activate plant immunity. Studies conducted by Stahl et al. and Ye et al. have demonstrated that indole, a plant organic volatile, can augment plant immunity to herbivorous insects (Stahl et al., 2016; Ye et al., 2019). Studies have revealed that MT **5** (**Figure 1**) can increase plant resistance to pathogens by activating *MAPK* pathways, resulting in the expression of numerous plant protection genes (Lee and Back, 2016a). To further exploit compounds as plant growth regulators and plant immune inducers, identifying targets, recognizing receptors, determining key activation points, and understanding activation mechanisms are necessary (Kusajima, 2019). An analysis of indole compounds about plant growth regulators and plant immune inducers is rarely documented. Therefore, this review examines the mechanism of action of indole compounds with regard to their application in the regulation of plant growth and activation of plant immunity. Our goal is to furnish a reliable source of knowledge for academics in related fields.

## Plant growth promoters

**Figure 2:**
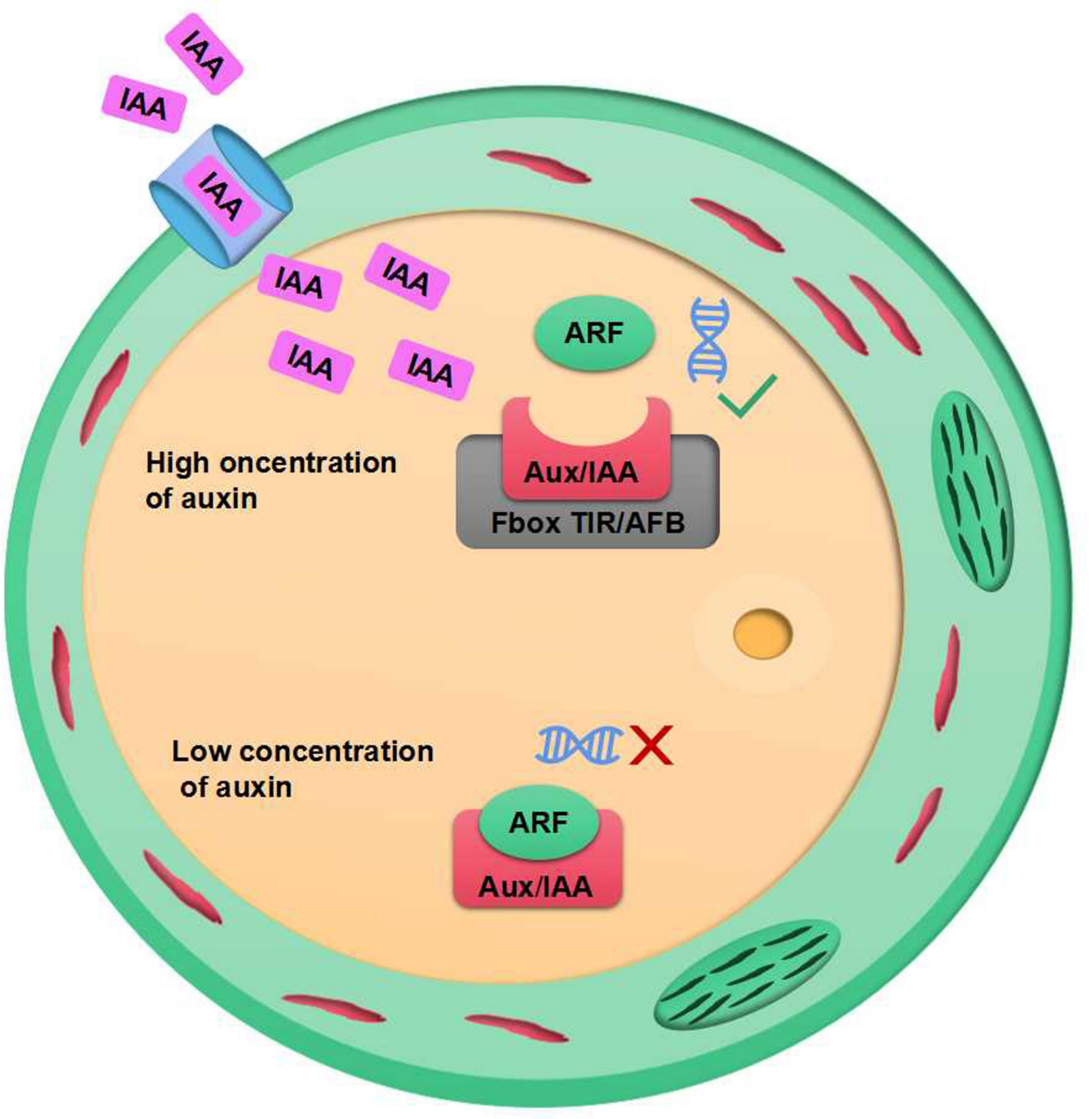
IAA signalling and gene expression system.

Plant growth promoters are a form of growth regulators that can encourage cell division, elongation, and the growth of vegetation, as well as the maturation of reproductive organs (Cai et al., 2020). The indole compounds with growth-regulating abilities are widespread. However, IAA is the most common and has a major impact on the growth and development of plants. IAA acts as a signal between rhizobium and plants. Experimental studies have demonstrated the application of *Stenotrophomonas maltophilia Sg3, Proteus mirabilis BjB17, Providencia rettgeri AlDp5, Bacillus thuringiensis TNJbx.3.3* and *Bacillus cereus GR12*, which are capable of synthesizing IAA, increased the number of pods of edamame beans (Zhang et al., 2022). The secretion of root in *Arabidopsis* can trigger *Falciphora oryza* to produce IAA, thus promoting the development of the lateral root of *Arabidopsis* (Sun et al., 2020). Gomes et al. and Zhang et al. (Gomes and Scortecci, 2021; Zhang et al., 2022) conducted reviews which revealed that IAA can modulate the transcription and expression of numerous genes through the ubiquitination complex, which is downstream of the repressor and activator of gene transcription factors. When there is a high growth hormone level in the cell, the ubiquitination complex is triggered by transport inhibitor response (TIR) proteins that are part of the growth hormone signaling pathway. This leads to the breakdown of Aux/IAA repressor molecules, thus allowing transcription factors to activate gene transcription in response to the growth hormone. At low growth hormone levels, cells tend to favor Aux/IAA due to dimer-mediated gene transcription by transcriptional auxin response factor (ARF) (as seen in **Figure 2**).

IBA (**Figure 1**) is a type of auxin structurally similar to IAA, with two methylene groups to its side chain (Dong et al., 2018; Damodaran and Strader, 2019). The indole ring of IBA is too elongated to successfully bind to the TIR1-Aux/IAA pocket, which is a necessary component of the peroxidase enzymes *IBR1, IBR3, IBR10*, and *ECH2* that are involved in the β-oxidation process leading to IAA production, resulting in a subsequent auxin-level signaling cascade (Fattorini et al., 2017; Aihebaier et al., 2019). It is yet to be determined whether IBA is an IAA-independent signaling molecule.

A new form of growth factor, indole-3-hexanoic acid (IHA) **6**, has been identified (illustrated in **Figure 1**). Structurally, it is analogous to IAA and IBA. It is derived from a novel pyridine carboxylate. It is recognized directly or indirectly by TIR1, the protein responsible for receiving signals from IHA, thus exhibiting a reaction similar to IAA (Napier, 2014). Studies have indicated that IHA can regulate the secretion of growth hormones by converting to IBA, and can also inhibit the transformation of IBA to IAA. Additionally, IHA has been found to induce responses that are distinct from IBA, such as increased amounts of *GH3.3* and *ACS4* (Song et al., 2021). However, the signaling process of IHA requires further exploration and study.

4-Chloro-indole-3-acetic acid (4-Cl-IAA) **7** (**Figure 1**) is a variant of IAA, which is distinguished by the presence of a chlorine atom at the 4-position of the indole ring. It was initially isolated from immature pea seeds (Marumo et al., 1968). However, in *peas*, only 4-Cl-IAA was able to stimulate gibberellin biosynthesis, inhibit the expression levels of ethylene biosynthesis genes (*PsACS4*, *PsACO2*, and *PsACO3*) in the pericarp, and upregulate the expression levels of ethylene receptor and signaling-related genes (*PsERS1*, *PsETR2*, *PsEBF1*, and *PsEBF2*) in the pericarp thereby reducing ethylene signaling output for pericarp growth (Jayasinghege et al., 2017). Reports indicate that 4-Cl-IAA is a critical signaling molecule in the aging process of *oat florets*, yet its precise mode of action remains uncertain (Dziurka et al., 2019). Generally, the distance between the aromatic ring and the carboxyl-terminal of IAA, IBA, 4-Cl-IAA, and other structurally similar growth factors is optimally within 0.55 Å for the most preferred activity (Cao et al., 2019; Damodaran and Strader, 2019). Research has shown that the activity of certain compounds in regulating plant growth is affected by the spatial configuration of the compounds. For example, Indole-3-succinic acid (ISA) **8** (**Figure 1**) proved to be more efficient than IAA or IBA in stimulating the growth of certain seedlings. Through chromatographic and diastereomeric crystallographic splitting, Daniel and his team were able to isolate the enantiomers *R*-(-)-ISA and *S*-(+)-ISA of ISA. It was determined that the plant growth-promoting activity of *R*-(-)-ISA was more effective than that of *S*-(+)-ISA (Armstrong et al., 2002).

**Figure 3:**
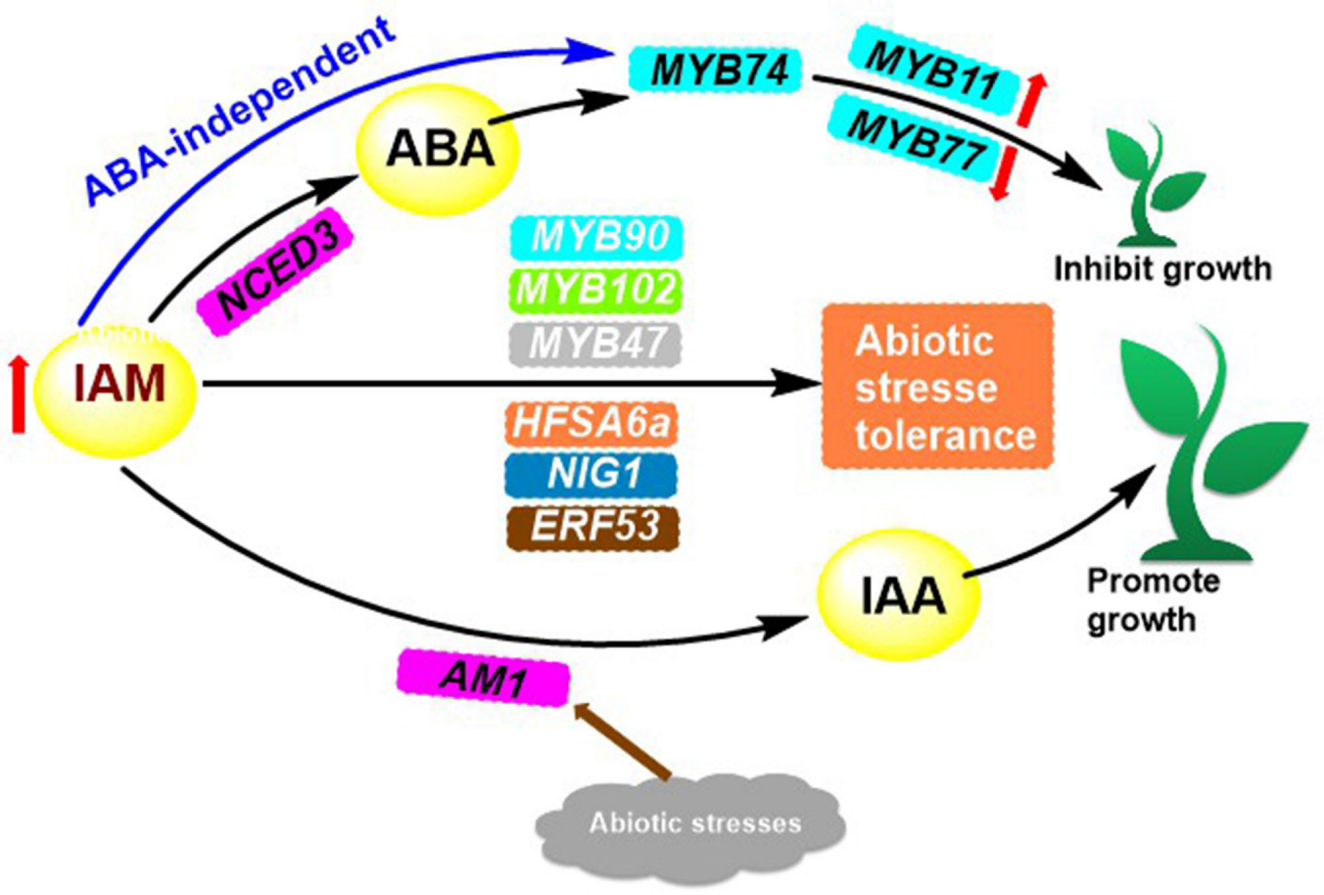
IAM accumulation-mediated transcriptional activation of MYB74 and its conversion to IAA regulates plant growth.

Indoleamine compounds are essential for the growth and development of plants and are involved in many significant biological processes. Such as stress response, growth and development, and reproduction. Indole-3-acetamide (IAM) **9** (**Figure 1**) is the precursor to the biosynthesis of IAA, which impacts plant growth through two pathways. Pathway 1 works towards the promotion of plant growth when IAM is converted to IAA by the specific hydrolase AMI1 (Pérez Alonso et al., 2020) (**Figure 3**). Pathway 2 is elucidated in depth through its inhibitory effects.

**Figure 4:**
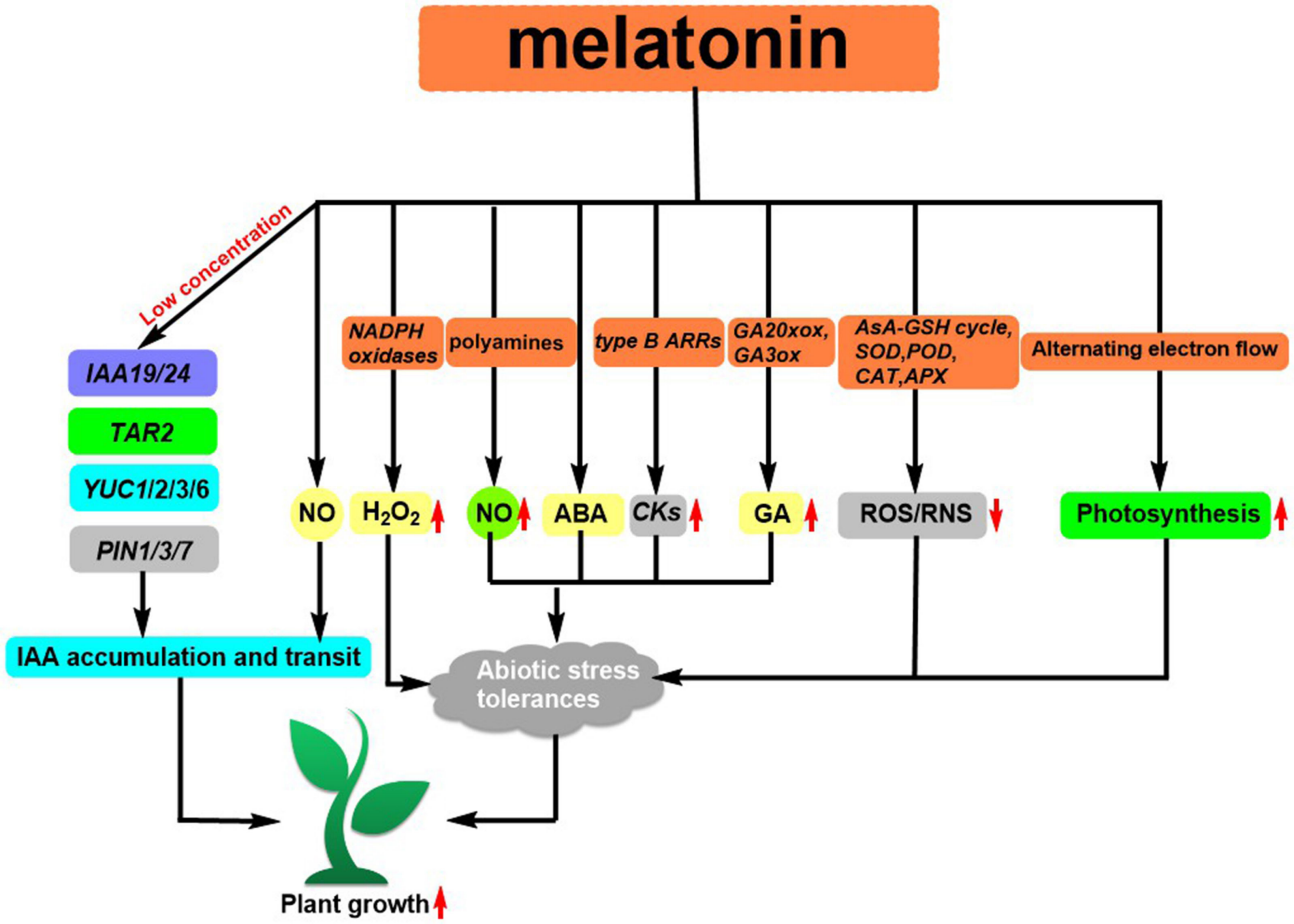
Melatonin regulates IAA biosynthesis and transport as well as regulates abiotic stress responses in plant.

As a major biosynthetic precursor, tryptophan (Trp) **10** (**Figure 1**) can help enhance the metabolites of *Clonostachys rosea*, resulting in a stronger capability to support the growth of tomato roots (Han et al., 2022). A class of ionic liquids (ILs) **11** (**Figure 1**) that demonstrate good solubility was developed by incorporating ammonium cations into the structure of L-Trp. The utilization of *Lettuce* increases its biomass by a range of 12-20% and enhances the uptake of certain nutrients (Szymaniak et al., 2021). Jasmonoyl-L-Tryptophan (JA-Trp) **12** (**Figure 1**) is a class of compounds that has the ability to disrupt AUX1, thus resulting in a failure of IAA. However, endogenous JA-Trp plays a minor role in the regulation of plant growth (Staswick et al., 2017). Additionally, Trp can be converted to melatonin by L-Trp decarboxylase (PSID) and tryptophan-5-hydroxylase (CYP71P1) (Zhang et al., 2022). This conversion has been found to have an effect on plant growth, such as promoting root growth after germination (Park and Back, 2012), influencing flowering time and regulating plant sugar metabolism (Zhao et al., 2015; Lee et al., 2019). The extent to which melatonin influences root elongation is dependent on the availability of IAA. At low concentrations, its ability to increase the expression of genes related to IAA signal transduction (*IAA19* and *IAA24*) and IAA biosynthesis (*YUC1*, *YUC2*, *YUC3*, *YUC6*, and *TAR2*) as well as some PIN proteins, has been demonstrated to facilitate lateral root development Auxin, coupled with its downstream signal nitric oxide, can activate the growth hormone signaling pathway (Wang et al., 2016; Wen et al., 2016), resulting in the production of adventitious roots in plants (Wen et al., 2016) (**Figure 4**). Zhang et al. reviewed (Zhang et al., 2022) that the first MT receptor in *Arabidopsis* was the candidate G protein-coupled receptor 2 (CAND2), a membrane protein that readily binds to MT (Wei et al., 2018). Research has demonstrated that the introduction of melatonin from an external source can induce the upregulation of the genes *RPOTm* and *RPOTmp* through the CAND2 receptor and its G protein alpha subunit (GP A1) (Bychkov et al., 2022). *Arabidopsis* Cand2/pmrt1, which is located at the plasma membrane, is known to interact with GPA1 and control stomatal movement by means of the NADPH oxidase-mediated reactive oxygen species (ROS) signaling pathway (Li et al., 2020). Recently, Zhao et al. reported that exogenous MT can promote the expression of *PITDC* and *PICOMT1* and increase the content of endogenous MT. And the endogenous MT can promote the expression of lignin biosynthesis-related genes (*PIPAL, PICCR, PICAD, PICOMT*, and *PIPOD*) and increases lignin accumulation, improving the strength of *Paeonia lactiflora Pall* stems (Zhao et al., 2022).

## Plant growth restrainers

Plant growth inhibitors are compounds, either man-made or natural, that impede the development of the entire plant or a particular part of the plant (Tuyen et al., 2018; Ellis et al., 2019).

An investigation into the biological activity of chemicals associated with root-parasitic plants revealed that IAA had a potent inhibitory effect on the seed germination of certain root-parasitic plants. Subsequent introduction of the 3-methylfuran-2(5*H*)-one structure into the carboxylic acid portion of IAA resulted in the formation of compound **13** (**Figure 1**), which was found to have dual activity, both inducing seed germination and suppressing the growth of embryonic roots after germination (Kuruma et al., 2021). The IAA analogs also showed significant inhibition of root growth in *Brassica napus.* In particular, compounds **14** and **15** inhibited up to 96% and 95% of *B. napus* roots at 100 mg/L, respectively, and persisted with 92% and 93% inhibition when the concentration was decreased to 10 mg/L (Wang et al., 2022). The conformational analysis demonstrated that the number of substituents on the benzene ring and electronic effects influenced the inhibitory action of *B. napus* roots. It was seen that CF_3_-substituted compounds were the most successful, and the presence of a long-chain alkyl group at the alpha position of the compounds increased their affinity for the TIR1 receptor. In addition, the benzene ring at the alkyl terminus facilitated the binding of the compounds to the TIR1 receptor **(** Hayashi, 2012; Wang et al., 2022 **)**. IAM (**Figure 1**), a precursor of IAA biosynthesis, has a bifurcated effect on plant growth. In pathway 1, it has a stimulatory effect, while in pathway 2, it has an inhibitory effect. This is caused by the increased levels of IAM in the plant, which leads to the expression of *NCED3*, a rate-limiting enzyme involved in the biosynthesis of abscisic acid (ABA). This, in turn, results in the overexpression of *R2R3 MYB* transcription factor genes *MYB74* or direct induction of *MYB74* overexpression, independent of ABA (Pérez Alonso et al., 2020; Ortiz García et al., 2022). Overexpression of *MYB74* has been observed to have an effect on certain genes associated with the proliferation of hyphal tissue cells (e.g., *MYB11*, *MYB77*), as well as genes related to the formation of lateral roots in plants, which ultimately leads to a decrease in plant growth (**Figure 3**).

**Figure 5:**
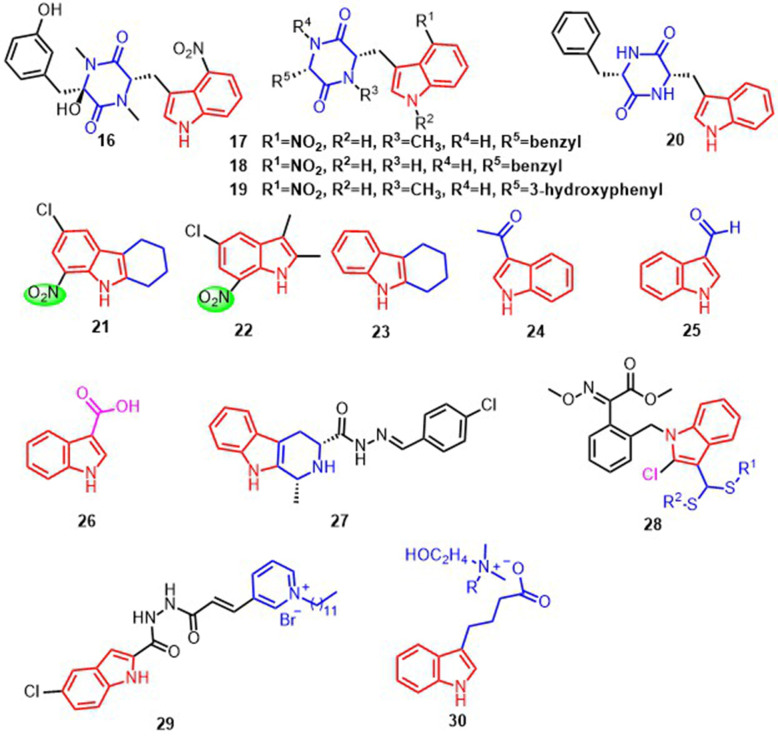
Structural formula of compounds 16-30.

Thaxtomins are a type of indole derivative featuring a 4-Nitroindole and diketopiperazine structure (King and Calhoun, 2009). Thaxtomin A **16** and thaxtomin C **17**, isolated from natural materials, the pre-emergence and post-emergence inhibitory activities against of *B. campestris* and *A. retroflexus* are more than 60%. And a study of such compounds by Zhang et al. found that compounds **16**, **17**, **18**, and **19** (**Figure 5**), with R^5^ as benzyl, showed significant inhibitory activity (≥85%) against *B. campestris* and *A. retroflexus* (King and Calhoun, 2009; Zhang et al., 2015). The nitro group at R^1^ is also critical for the growth inhibition of *B. campestris* and *A. retroflexus*. For example, compound **20** (**Figure 5**) with the nitro removed exhibited only 10% pre-emergence inhibition activity against *B. campestris* and *A. retroflexus*. In addition, the benzyl portion on R^5^ and the hydroxyl group on the diketopiperazine structure affect the crop selection properties of such compounds. Protoporphyrinogen oxidase (PPO) may be a potential target for compounds **16**, **17**, **18**, and **19**, which indirectly affect chlorophyll synthesis and inhibit plant growth (Duke et al., 2019). Another class of compounds with 7-Nitroindole structure, **21** and **22** (**Figure 5**), can reduce the dry biomass of the weeds of *Ipomoea grandifolia* and *Senna alata* by 40% and 37%. This leads to a decrease in plant ATP synthesis and CO_2_ fixation, interfering plant development (de Souza et al., 2020). In addition, the introduction of methylene structure can improve the lipophilicity of these compounds and promote their entry into plant cells to exert inhibitory effects. For example, compound **23** (**Figure 5**) inhibited seed germination and root length of plants by 22% and 49.6%, respectively.

The introduction of the methyl ketone structure at the indole 3-position of compound **24** (**Figure 5**) produced a considerable inhibition of germination and shoot growth of the seeds of *Amaranthus tricolor* (Chotpatiwetchkul et al., 2022). At a concentration of 400-800 μM, the germination of seeds was completely inhibited. Compound **24** demonstrated inhibitory effects against hydroxyphenylpyruvate dioxygenase (HPPD), potentially interrupting the transformation of HPP to homogentisate and subsequently impeding the formation of tocopherols and plastoquinones. This disruption in the production of carotenoids may result in abnormal plant growth or death (Ndikuryayo et al., 2017; Chotpatiwetchkul et al., 2022). The conformational analysis showed that replacing the 7-position of the indole ring in compound **24** with C to N could enhance the inhibitory activity against HPPD (Chotpatiwetchkul et al., 2022).

## Biological stress resistance

Biological stress is a general term for various biological factors that are unfavorable to plant survival and development. It is usually caused by infection and competition, such as diseases, pests, weed hazards, etc (Moustafa-Farag et al., 2019).

Indole can serve as signals for some chewing insect infestations or for necrotic pathogens to invade plants. Studies have found that the indole biosynthesis rate in *maize* and *rice* quickly increases when exposed to herbivorous insect attacks. Indole has been demonstrated to bolster plant immunity when faced with pathogenic threats by prompting the build-up of H_2_O_2_, which activates the MAPK cascade and phosphorylates protein-like transcription factors. This leads to the activation of defense genes (Jalmi and Sinha, 2015; Mittler and Blumwald, 2015; Perez and Brown, 2015; Shen et al., 2018; Ye et al., 2019), including JA and plant antitoxin biosynthesis genes, cure-associated proteins, and antioxidant enzymes (Gozzo and Faoro, 2013; Shen et al., 2018). In *Camellia sinensis*, indole is the expression of early defense genes involved in Ca^2+^ signal, MPK signal, and JA biosynthesis, and the production of secondary metabolites associated with JA and defense is initiated, thus increasing the resistance of *Camellia sinensis* to herbivores (Ye et al., 2021).

**Figure 6:**
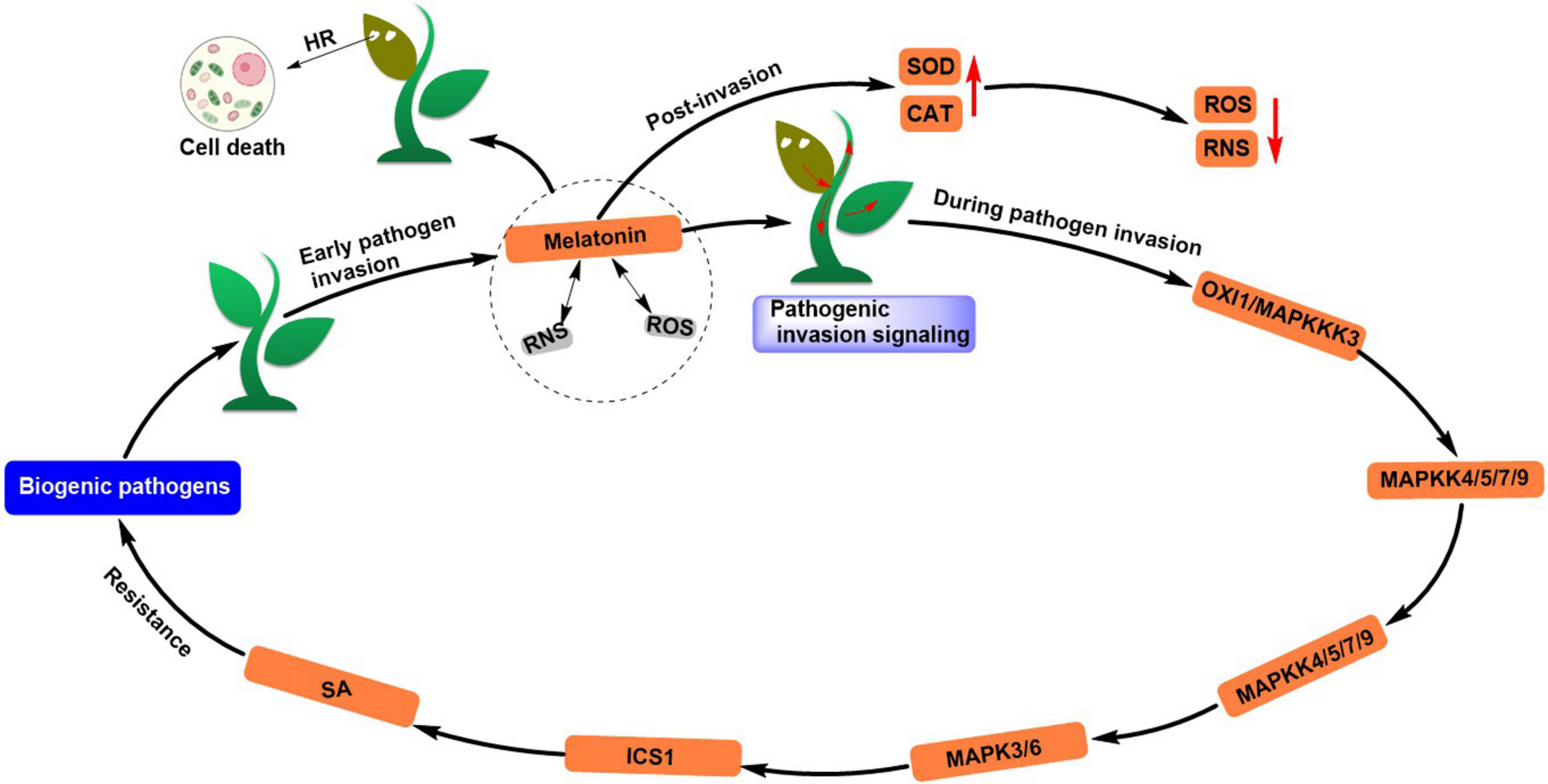
Melatonin induces resistance to biotic stress responses in plants.

MT also plays a critical role in enhancing plant resistance to biotic stresses. Zhao et al. reviewed (Zhao et al., 2020) that MT, together with ROS and reactive nitrogen species (RNS), promotes cell death and prevents pathogen invasion by forming an integrated feedforward loop during the early stages of pathogen invasion (Gaupels et al., 2017; Arnao and Hernandez-Ruiz, 2018). In addition, the MT-ROS-RNS composition transmits pathogen invasion signals from the starting site to the entire plant and confers plant biological tolerance early in infection. During pathogen invasion, MT acts upstream of SA and accumulates it, and SA further mediates immune response dependent on *MAPK* signaling cascade. Moreover, MT may also improve plant immunity by altering cell wall composition and influencing crosstalk between auxin and JA signaling pathways. MT further removes excess ROS and RNS by activating gene expression of antioxidant enzymes (SOD, H_2_O_2_, etc.) and promotes redox homeostasis in plant systems (Reiter et al., 2009; Arnao and Hernández-Ruiz, 2019) (**Figure 6**). MT increased early in pathogen invasion and was restored to normal levels by expression of metabolic genes (*IDO* or *2-OGDD*) (Tan et al., 2007; Lee et al., 2016b; Yu et al., 2018).

The compounds indole-3-formaldehyde **25** and indole-3-carboxylic acid **26** (**Figure 5**) extracted from *Purpureocillium lilaci-num* had better immune activation for some plants infected by the *tobacco mosaic virus* (TMV). The application of **25** and **26** can increase the level of transcription of *Nonexpresser of PR1* (*NPR1*), *pathogenesis-related 1* (*PR1*), *pathogenesis-related 2* (*PR2*), *pathogenesis-related 5* (*PR5*) and *phenylalanine ammonia-lyase(PAL)*, **25** and **26** can also upregulate the activity of defensive enzymes such as *catalase* (*CAT*) and *peroxidase* (*POD*) to reduce peroxide damage to membranes. In addition, **25** also improves (*PAL*) activity and transcription levels of *isochorismate* (*ICS*) and *avrPphB susceptible 3* (*PBS3*) to facilitate SA accumulation. But **26** only mediates SA accumulation through the *PAL* pathway, triggering systemic acquired resistance in plants (SAR) (Sun et al., 2022). A class of compound **27** (**Figure 5**) reported by Wang et al. was also able to induce SA and *PR2* expression and improve plant resistance to TMV by activating reactive oxygen species and antioxidant levels (Wang and Song, 2020).

**Figure 7:**
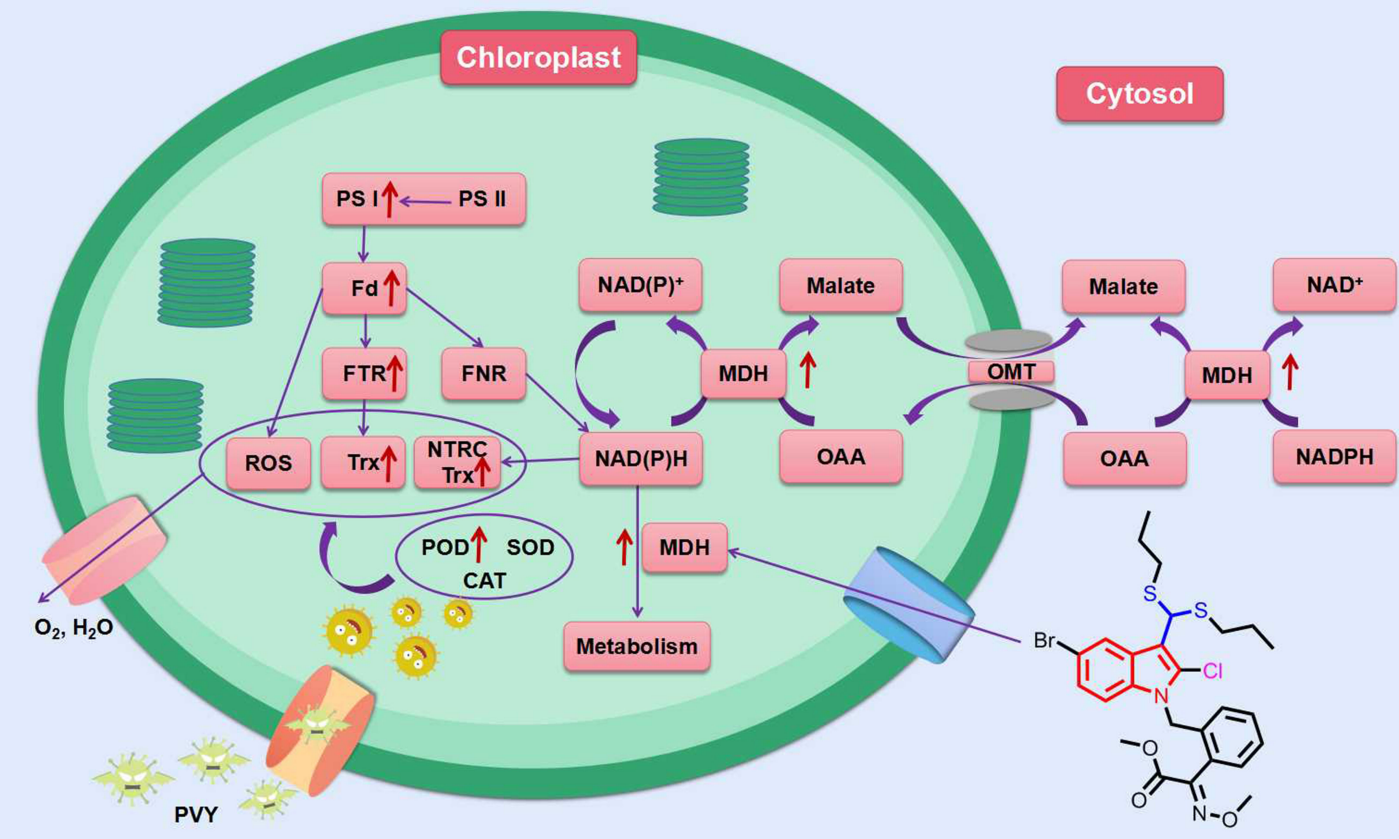
MDH signaling pathway in tobacco response to compound 28. Red arrows indicate that the protein is upregulated in this pathway. (Fd, ferredoxin; FNR, ferredoxin-NADP reductase; FTR, ferredoxin-thioredoxin reductase; MDH, malate dehydrogenase; NTRC, chloroplast NADPH-thioredoxin reductase; OAA, oxaloacetate; OMT, malate/OAA translocators; PS I, photosystem I; PS II, photosystem I; ROS, reactive oxygen species; Trx, thioredoxin). (Wei et al., 2019).

In addition to the above indole compounds that enhance plant resistance to viruses *via* the SA pathway, Wei et al. reported that compound **28** (**Figure 5**) with a disulfide structure can promote photosynthesis by enhancing chlorophyll content, and also can enhance plant resistance to TMV, cucumber mosaic virus (CMV) and potato Y virus (PVY) by enhancing the activities of defense enzymes such as SOD, POD, PAL and CAT. Futhermore, compound **28** was able to increase malate dehydrogenase (MDH) activity and act with MDH signaling pathway (**Figure 7**) (Wei et al., 2019).

**Figure 8:**
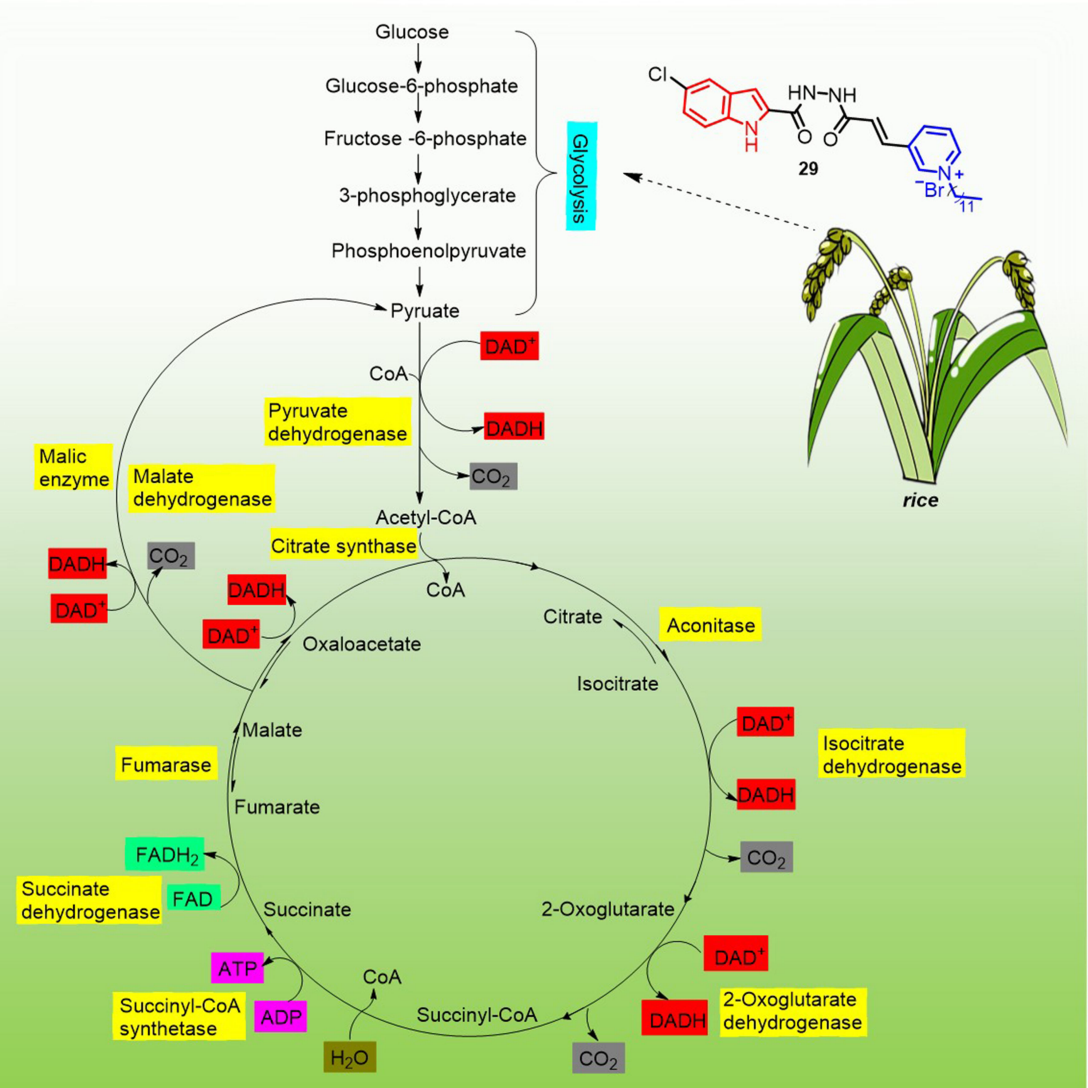
Mechanism of disease resistance triggered by compound 29 stimulation in *rice*.

In 2022, Li et al. reported that indole derivatives **29** (**Figure 5**) containing pyridinium salts could regulate the conversion of glycolysis in *rice* to produce pyruvate, which was further decarboxylated to produce acetyl-CoA and subsequently entered the citric acid cycle where NAD^+^ was reduced to NADH. The NADH produced by this process was fed into the oxidative phosphorylation way (Li et al., 2022a). The result of the two closely linked ways improves plant resistance to *Xanthomonas oryzae* pv. *oryzicola* and *X. oryzae* pv. *oryzae*. by oxidizing nutrients and generating available chemical energy to give to the plant (**Figure 8**).

## Abiotic stress resistance

Abiotic stress is the result of an abiotic factor on a plant in a given environment, which can disrupt its growth and development processes (Mittler, 2002).

IAA, one of the most abundant phytohormones in plants, not only promotes root growth but also enhances plant resistance to abiotic stresses. Studies have shown that the external application of IAA can significantly increase the activities of POD and SOD, as well as the contents of chlorophyll, carotenoid, and soluble protein in *Cyphomandra betacea* seedlings. Furthermore, it can reduce the Cd content in different organs and improve the resistance of plants to Cd (Li et al., 2022b). Salt stress in plants led to an overexpression of growth hormones, which manifested in increased root hair formation. This alteration augmented the capacity of plants to take up water during the drought (Germanà et al., 2015). IBA, was found to be effective in counteracting the inhibitory effects of Cd and mannitol on plant adventitious roots, and it was also successful in restoring the levels of soluble proteins that had been reduced due to Cd and mannitol (Li et al., 2018). Pernak et al. reported a class of ILs **30** composed of alkylated choline cations and IBA anions that exhibit excellent physical properties such as hydrophobicity and surface activity (Kaczmarek et al., 2020). Compound **30** were found to promote the uptake of essential material nutrients (P, K, Ca, Mg, Na, and Mn) by lettuce, while hindering the uptake of Fe, Zn, and Cu, resulting in a 20% increase in lettuce biomass production. However, the exact mechanism of action is yet to be determined. Additionally, IAM, as a precursor of IAA, can improve plant abiotic stress tolerance by enhancing the expression of abiotic stress-related genes, such as *NIG1* and *MYB47* (Kim and Kim, 2006; Ding et al., 2013).

Abiotic stress can produce a large amount of ROS and RNS in plants, resulting in oxidative damage to plant cells (Mittler, 2002). MT has been identified to possess antioxidant properties, which can stimulate the activity of antioxidant enzymes such as the Ascorbate-glutathione (AsA-GSH) cycle, SOD, POD, CAT, APX, and the expression of related genes. This helps to eliminate excess ROS and RNS, improving the resilience of plants to abiotic stresses (Shi et al., 2014; Li et al., 2016; Marta et al., 2016; Cui et al., 2017). MT can help to increase abiotic stress resistance through its downstream signals H_2_O_2_ and NO. For instance, under low-temperature stress, MT can inhibit sulfhydryl nitrosylation activity and promote NADPH oxidase activity to generate H_2_O_2_, protecting against low-temperature stress (Gong et al., 2017). In Fe-deficient plants, MT regulates the plant by modulating the polyamine-induced NO production (Zhou et al., 2016). Fu et al. revealed that MT has the potential to act as an antecedent to ABA, thereby regulating the plant’s response to low-temperature stress. Additionally, MT is known to assist plants in dealing with abiotic stresses (Arnao et al., 2018). Under heat stress, MT was able to up-regulate the expression of cytokinin (CKs) synthesis genes and their transcription factors *type B ARRs* (Zhang et al., 2017). Under salt stress, melatonin induced the expression of gibberellin (GA) synthesis genes *GA20xox* and *GA3ox* (Zhang et al., 2014a). In addition, MT resists the inhibitory effect of abiotic stress on plant photosynthesis by regulating photosynthetic carbon reduction, photorespiration, and O_2_-dependent alternate electron flow balance (Zhao et al., 2016; Chen et al., 2017; Li et al., 2017) (**Figure 4**).

## Conclusion and perspectives

Investigating the role of indole compounds in the process of plant growth regulation, as well as their impact on plant resistance to both biological and abiotic stress, is the main focus of this review. The promotion of plant growth by indole analogs is closely related to IAA. For instance, IBA requires β-oxidation to form IAA, while IAM can be converted to IAA with the help of a specific hydrolase (AM1). Furthermore, melatonin is essential for enhancing IAA-related transduction genes, biosynthetic genes, and some PIN proteins, thus aiding in the development of plant roots. Indole compounds can boost plant resistance to various biotic stresses through direct or indirect action on SA, JA, and MDH pathways and increase the activity of associated defense response enzymes. Research has revealed that **26** and **27** can heighten plant defenses against TMV by augmenting the activity of defensive enzymes like CAT and POD and stimulating salicylic acid accumulation. Melatonin is the initial factor that triggers the increase of SA, which then activates the MAPK signaling cascade to regulate the immune response. In addition, melatonin can also resist the adverse effects of salt, drought, and cold on plants by promoting the activity of various antioxidant enzymes and the expression of related genes.

Numerous indole compounds have been observed to influence plant growth and stress tolerance. Yet, how these signals are detected by the plant and amplified for further regulation of development and stress resistance is largely unknown. Subsequent studies should focus on examining the effects of indole analog signals on biotic and abiotic stress signal receptors and how they may intensify the signal transduction process. Investigating the interplay between indole signals in plants and other phytohormones will be advantageous in comprehending the mechanism of indole compounds in regulating plant growth and resilience to stress.

## Author contributions

All authors have read and agreed to the published version of the manuscript. PS collected and analyzed the refences, wrote the draft of the manuscript. PS, YH and AL completed the Figures. XY and JW reviewed and edited the manuscript. All authors contributed to the article and approved the submitted version.
